# Familial Mediterranean Fever in Spain: Time Trend and Spatial Distribution of the Hospitalizations

**DOI:** 10.3390/ijerph20054374

**Published:** 2023-02-28

**Authors:** Elisa Gallego, Greta Arias-Merino, Germán Sánchez-Díaz, Ana Villaverde-Hueso, Manuel Posada de la Paz, Verónica Alonso-Ferreira

**Affiliations:** 1 Instituto de Investigación de Enfermedades Raras (IIER), Instituto de Salud Carlos III, 28029 Madrid, Spain; 2 Escuela Internacional de Doctorado, Universidad Nacional de Educación a Distancia (UNED), Calle Bravo Murillo, 38, 28015 Madrid, Spain; 3 Centro de Investigación Biomédica en Red de Enfermedades Raras (CIBERER), 28029 Madrid, Spain

**Keywords:** Familial Mediterranean Fever, rare disease, health information system, population-based study, hospitalizations

## Abstract

Familial Mediterranean Fever (FMF) is a rare, hereditary, auto-inflammatory disease. The aims of this study were to explore the time trend and geographical distribution of hospitalizations in Spain from 2008 to 2015. We identified hospitalizations of FMF from the Spanish Minimum Basic Data Set at hospital discharge, using ICD-9-CM code 277.31. Age-specific and age-adjusted hospitalization rates were calculated. The time trend and the average percentage change were analyzed using Joinpoint regression. Standardized morbidity ratios were calculated and mapped by province. A total of 960 FMF-related hospitalizations (52% men) were identified across the period 2008–2015, with an increase in hospitalizations of 4.9% per year being detected (*p* < 0.05). The risk of hospitalization was higher than expected for the national total (SMR > 1) in 13 provinces (5 in the Mediterranean area), and lower (SMR < 1) in 14 provinces (3 in the Mediterranean area). There was an increase in hospitalizations of patients with FMF in Spain throughout the study period, with a risk of hospitalization that was higher, though not exclusively so, in provinces along the Mediterranean coast. These findings contribute to the visibility of FMF and provide useful information for health planning. Further research should take into account new population-based information, in order to continue monitoring this disease.

## 1. Introduction

**Figure 1:**
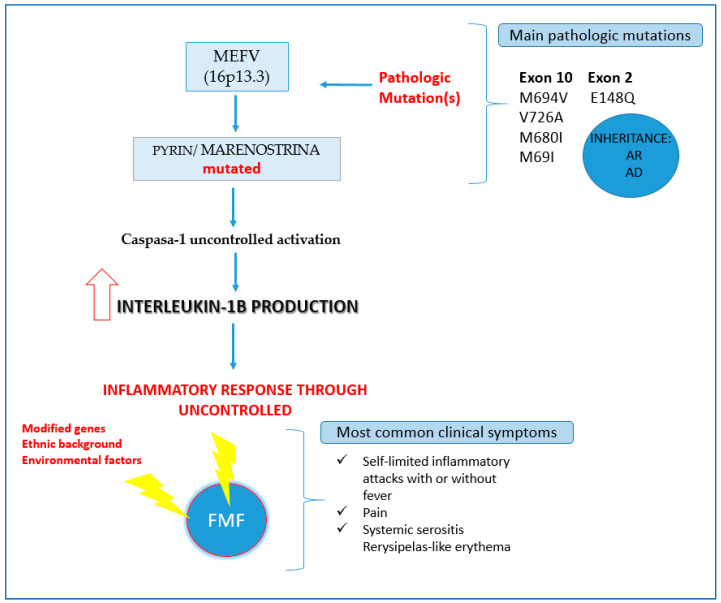
Inheritance of FMF and pathology associated with elevated IL-1. Elaborated by the authors.

Familial Mediterranean Fever (FMF) is an auto-inflammatory, monogenic disease, generally of autosomal recessive inheritance. It is due to mutations in the *MEFV* (MEditerranean FeVer) gene, present on the short arm of chromosome 16. This gene encodes a protein named pyrin, which has a regulatory function of inflammation. It is characterized by recurrent and self-limited periodic episodes of fever, pain, polyserositis, synovitis, and dermatological manifestations [1,2,3,4,5,6]. The inheritance of FMF and the pathology associated with elevated IL-1 are shown in Figure 1. The severity and frequency of the symptoms is variable, since the presentation of the disease depends on environmental and genetic factors. The main cause of hospitalization and mortality is secondary amyloidosis, the risk of development of which depends on ethnicity, genotype, and treatment [4].

FMF is characterized by great ethnic and geographic variability [1]. Indeed, its epidemiology is changing rapidly, due to migrations and enhanced diagnostic sensitivity. Currently, it has a global distribution, with an estimate of over 200,000 people affected worldwide [4,5,7,8]. Its origins can be traced to populations from the eastern Mediterranean, where the frequency of carriers of the *MEFV* gene in the general population is 1 in 3–7 inhabitants and the prevalence of the disease ranges from 1 case per 150–1000 inhabitants [4,5,6,9]. Differences between the frequency of mutation carriers and the prevalence of the disease show that penetrance is incomplete and differs between populations [5]. Turkey is probably the country with the highest number of people affected, with an absolute frequency of 70,000–100,000 [5]. Due to its prevalence of less than 5 cases per 10,000 inhabitants, FMF is considered a rare disease in the European Union [10]. It is more frequent in countries located in the south-east of Europe, such as Greece, Cyprus, and Italy [6,11,12]. In France, 5000 to 10,000 patients are estimated to be diagnosed with FMF [13]. Lainka et al. estimated the incidence of FMF in Germany as 3 per million person-years for the entire population of children and 55 per million person-years for children of Turkish descent [14].

In Spain, four cases belonging to two different families were reported for the first time in 1962 [15]. In 1988, a group of patients with FMF was identified among the so-called ‘Chuetas’, a community made up of descendants of converted Jews from the island of Majorca (carrier frequency of 1:1000). *MEFV* gene mutations were confirmed to match those described in the Sephardic Jewish population [16,17,18]. Aldea et al., who studied FMF in a cohort of 55 patients with the objective of ascertaining their clinical and genetic characteristics, described a carrier frequency of 2.5% and a prevalence of 1: 60,000–120,000 [19]. In addition, isolated cases of patients with FMF have been reported in different regions of Spain [15,20,21,22].

Due to the increase in prevalence reported in Europe [6,23], it is essential to have population-based information to monitor this disease, increase knowledge about it, and improve its visibility, especially in Mediterranean countries, such as Spain. To date, however, this disease has not been included in Spain’s population-based Rare Disease Registry (Registro Estatal de Enfermedades Raras/ReeR) [24]. Currently, the most complete source assessing the FMF morbidity profile is the Minimum Basic Data Set (MBDS) [25], which is an administrative record of all hospital admissions nationwide.

The aim of the study was to explore the time trend and geographical distribution of FMF hospitalizations in Spain from 2008 to 2015, by analyzing the MBDS case reports.

## 2. Materials and Methods

Data were sourced from hospital discharges recorded in the Spanish National Health System (MBDS). In addition to discharges or the number of hospital admissions, the MBDS provides other variables of interest, including year, sex, age, main and secondary diagnoses, average hospital stay, department of admission, type of discharge, and province.

FMF has been registered in the MBDS since 2008 under code 277.31 “Familial Mediterranean Fever” of the International Classification of Diseases, Ninth Revision, Clinical Modification (ICD-9-CM). In 2016, the coding was changed to the ICD-10-CM Spanish version (ICD-10-ES), with this disease appearing under the headings “Non-neuropathic heredofamilial amyloidosis” (E85.0 in 2016 and 2017) and “Periodic fever syndromes” (M04.1 since 2018). In order to explore a more uniform time series and avoid the possible influence of coding changes, we studied the period of 2008–2015, retrieving all cases compatible with code 277.31 in any diagnostic position.

The characteristics of FMF-related hospitalizations were analyzed by sex. Age-specific hospitalization rates and their distribution by sex were calculated. The age-adjusted hospitalization rates for each year were also analyzed, taking the standard European population as a reference. All rates were expressed per million inhabitants, with Spanish population data being drawn from the National Statistics Institute (Instituto Nacional de Estadística) [26].

The time trend and average percentage change (APC) were analyzed using Joinpoint regression. For geographical analysis purposes, the standardized morbidity ratios (SMRs) were calculated by province, taking the Spanish population as reference (SMR = 1.00). Provinces that displayed values significantly lower or higher than expected were identified by their 95% confidence intervals (95% CIs). For better visualization of the results, any variability found was represented cartographically.

All analyses were performed with the STATA v.17, R version 4.2.0, Epidat v.4.2, and Joinpoint v.4.7 statistical software packages, and cartographical representations with the QGIS v.3.12 program.

## 3. Results

In the period from 2008 to 2015, 960 hospital admissions with a diagnosis of FMF were recorded, with this disease being the main diagnosis in 42.8% of discharges. Men represented 52.3% (502) and women 47.7% (458) of cases, with a male–female ratio of 1.1. The age range was 0 to 93 years, and the median age was 33 years in men (IQR: 11–59) and 27 in women (IQR: 13–47), with this difference being significant (*p* < 0.05); 31.1% of patients were under 15 years old and only two cases were under one year old. The median hospital stay was 5 days (IQR: 2–9), similar in both sexes (*p* = 0.676). A total of 18.9% of the registered hospitalizations were readmissions, with no differences by sex (chi2: 0.22; *p* = 0.641).

Table 1 shows the distribution of hospital admissions, with a breakdown by department, type of discharge, and sex. The departments with the highest number of admissions were internal medicine, pediatrics, nephrology, digestive, surgery (general and digestive), and rheumatology. 

Table 2 shows the main diagnoses recorded, classified into large groups of diseases according to the ICD-9-CM. FMF was the main cause of hospitalization in 411 discharges.

With regard to impaired kidney function, 8.7% of discharges had chronic renal failure, 4.9% tubulointerstitial nephritis, 3.5% acute renal failure, and 2.7% nephrotic syndrome; 2.9% were on renal dialysis and 1.6% had undergone a kidney transplant. In all, 3.8% of hospitalizations presented unspecified amyloidosis.

According to the characteristic symptoms or signs of the FMF episodes, the most frequent diagnoses were abdominal pain (9.0% of hospitalizations), fever (5.0%), arthritis (4.8%), non-infectious pericarditis (2.4%), pleurisy (2.0%), arthralgias (2.0%), myalgias (1.2%), chest or pleural pain (0.7%), sacroiliitis (0.2%), and peritonitis (0.1%). Among the auto-inflammatory diseases that are part of the differential diagnosis of FMF, the following comorbidities were noteworthy: juvenile rheumatoid arthritis (34 discharges; 3.5%), rheumatoid arthritis (24; 2.5%), Behçet’s disease (10; 1.0%), panarteritis nodosa (8; 0.8%), seronegative arthropathies, such as psoriatic arthritis and ankylosing spondylitis (6; 0.6%), and systemic lupus erythematosus (6 discharges; 0.6%).

**Figure 2:**
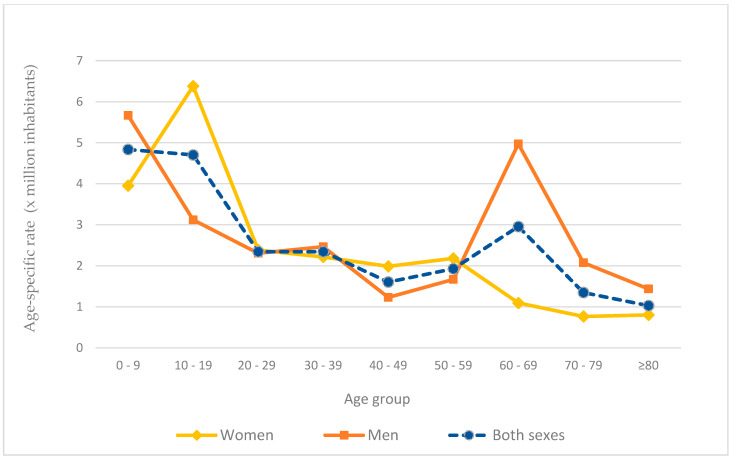
Age-specific hospitalization rates of patients with FMF in Spain by sex, 2008–2015.

The age-specific hospitalization rate was higher from ages 0 to 9 years in both sexes, with 4.84 per million inhabitants (5.67 in men; 3.95 in women). Figure 2 shows that the highest rate in women was found in adolescents between 10 and 19 years old. In men, in addition to the 0- to 9-year age group, the 60–69 age group stands out (4.97 per million inhabitants).

**Figure 3:**
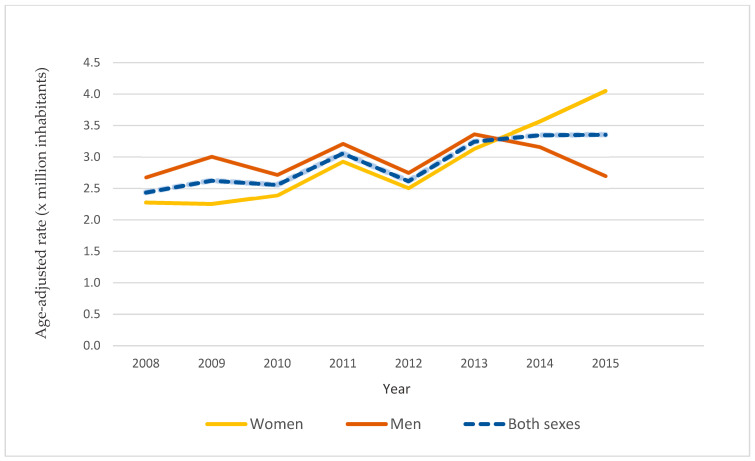
Age-adjusted hospitalization rates of patients with FMF in Spain, 2008–2015.

Throughout the period studied, the age-adjusted hospitalization rates for both sexes ranged from 2.47 (95% CI: 0.70–4.73) per million inhabitants in 2008 to 3.35 (95% CI: 1.29–5.41) per million in 2015 (Figure 3). For both sexes, the average annual increase is 4.9% per year (95% CI: 2.20–7.63; *p* < 0.05). The rate among women was 2.28 per million (95% CI: 1.56–2.99) in 2008 and 4.05 (95% CI: 3.11–4.49) in 2015, showing a rising trend in cases (APC = 8.67%, 95% CI: 5.2–12.3; *p* < 0.05). In the case of men, the rate varied from 2.68 per million inhabitants (95% CI: 1.97–3.39) to 2.70 per million (95% CI: 1.96–3.43), though this variation was not significant (*p* = 0.890).

**Figure 4:**
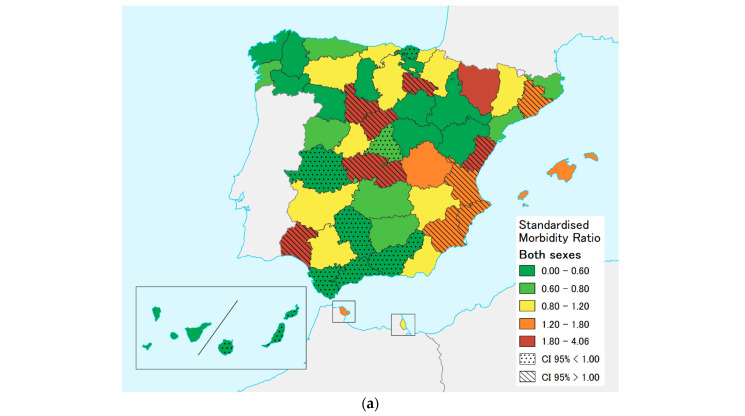

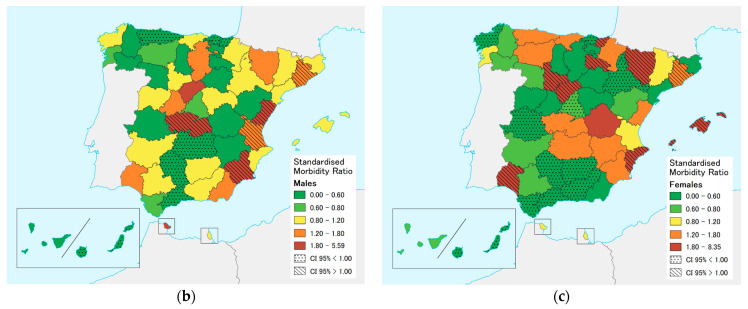
Geographical variability of FMF hospitalizations in Spain (2008–2015): Standardized morbidity ratios (SMRs) and 95% confidence intervals are highlighted in those cases where they were higher (striped) or lower (dotted) than expected. (**a**) Both sexes; (**b**) males; (**c**) females.

Table 3 shows provinces that recorded values that were significantly lower or higher than expected. Of the 52 Spanish provinces and autonomous cities, 13 (25.0%) registered a higher-than-expected risk and 14 (26.9%) a lower-than-expected risk of hospitalization. Of the 14 regions in the Mediterranean area, 5 (35.5%) displayed a higher risk and 3 (21.4%) a lower risk. Higher-than-expected hospitalization rates were found for both sexes in 10 provinces: Alicante, Barcelona, Castellón, Huelva, La Rioja, Murcia, Segovia, Toledo, Valencia, and Valladolid (SMR > 1). Conversely, rates were lower than expected in Cáceres, Cadiz, Cordoba, Granada, Madrid, Malaga, Las Palmas, and Vizcaya (SMR < 1). This geographical variability, along with the breakdown for men and women separately, are depicted in Figure 4.

## 4. Discussion

This is the first study to make an approximation of the characteristics of the population with FMF in Spain, along with its temporal and geographical variability, pinpointing the provinces with the highest rates of hospitalization. A lower proportion of admissions is shown among women than among men; while this finding is in line with what has been reported in some countries, mainly those situated in Eastern Mediterranean regions, such as Anatolia (Turkey) or Lebanon, where a higher prevalence of FMF male patients have been reported [27,28,29], most describe a similar disease distribution across the sexes [1]. The mean age of hospitalized patients likewise agrees with what has been reported by similar studies [30,31]. FMF usually manifests before the age of 20 years in 90% of cases, and during the first decade of life in 60% of cases [4], a finding consistent with the high percentage of hospitalizations of patients under the age of 20 [28]. At an early age in life, crises usually present as episodes of fever without signs of serositis, which makes it difficult to identify this disease correctly [32,33,34], causing delays in diagnosis, estimated to be around 5 years in the pediatric population and 13 years in the case of adults [30,35].

The greater number of admissions to internal medicine as compared to other hospital departments is accounted for, firstly, by the systemic characteristics of the disease, and secondly, by the most common symptoms, such as episodes lasting 3–4 days, consisting mainly of high fever (more than 38 °C), abdominal pain, arthritic joint pain, and chest pain [1,4]. The high number of hospitalizations in the pediatric department would presumably be related to the fact that episode severity and frequency is usually greater in the infant stage [1]. Nephrology, too, was one of the departments to show the highest number of admissions, since morbidity is mainly related to what has been recorded (hematuria, proteinuria, pyelonephritis, glomerulonephritis) and/or the development of secondary renal amyloidosis, which presents with nephrotic syndrome and chronic renal insufficiency [36,37].

Diagnosis remains primarily based on clinical findings (episodes of intermittent fever with concomitant serositis, in the absence of an alternative cause), response to colchicine, ethnicity, and family history [38,39]. The most widely used criteria for the diagnosis are based on Tel HaShomer, Livneh et al., pediatric criteria, and the new Eurofever/PRINTO classification for FMF. The Tel HaShomer and Livneh et al. criteria originated from countries where the prevalence of FMF is very high. The diagnosis of FMF should be considered when a patient of Mediterranean origin meets two major criteria, or one major and two minor criteria [4,40]. Most recently, adult and pediatric rheumatology experts have created a new set of classification criteria (Eurofever/PRINTO classification criteria) combining clinical manifestations with genotype for the first time [34,38]. FMF can easily be confused with other auto-inflammatory syndromes that present with periodic fever, such as factor receptor-associated periodic syndrome, cryopyrin-associated auto-inflammatory syndrome, and hyperimmunoglobulin D syndrome [23]. Alternatively, they can share symptoms or appear associated with vasculitis, such as Schönlein–Henoch syndrome, panarteritis nodosa, or Beçhet’s disease, so it is essential to make an optimal differential diagnosis with such entities [40,41]. Other causes of morbidity associated with FMF are intestinal obstructions, abdominal pain, peritoneal adhesions, arthritis, sacroiliitis, or colchicine toxicity [1,39].

The development of secondary amyloidosis (AA amyloidosis) has been described as the sole factor that significantly contributes to increased morbidity and mortality in patients not controlled with treatment [36]. AA amyloidosis due to amyloid A deposit occurs mainly in the kidney, although amyloid deposits in the intestine, thyroid, spleen, liver, or adrenal glands have also been described. FMF amyloidosis is a secondary reactive amyloidosis characteristic of chronic inflammatory diseases in which amyloid deposition is associated with elevated serum amyloid A protein values [1,4]. In cases in which the only manifestation is amyloidosis, the use of genetic tests for correct diagnosis becomes important.

### 4.1. Temporal Variability

In recent years, new anti-IL1β1 biological drugs have been developed. These are used in patients who do not respond well to colchicine, and reduce the risk of amyloidosis, comorbidities, and mortality associated with the disease [2]. 

There is no consensus on a specific definition of ‘colchicine resistance’. However, the presence of recurrent clinical attacks and persistent elevated inflammation markers in a patient receiving the maximum tolerated dose of colchicine led to that diagnosis [3]. Even so, an increase in hospitalization rates was found across the study period. Although one cannot rule out that this increase in hospital morbidity might be related to an increase in prevalence, it could also be accounted for by greater knowledge of the disease on the part of physicians and by the inclusion of FMF among the differential diagnoses of auto-inflammatory diseases that present with fever. Furthermore, it may be due to the better registration and coding of discharges, the development of more sensitive and specific genetic tests, and the creation of specialized hospital units. Increased immigration from countries where FMF is a common disease could also influence this result.

### 4.2. Geographic Variability

Increased hospitalization rates were detected in several provinces along the Mediterranean coast. The M694V mutation, which is associated with severe disease, has been described as the most prevalent in the Balearic Island ‘Chueta’ community [4,15,16,19]. Hence, higher overall hospitalization rates were expected in this province. Yet, it was only in the case of women that the risk of hospitalization was seen to be higher.

The interprovincial variability could be due to the distribution of certain factors, such as the different mutations of the *MEFV* gene, environmental factors, or the country of origin of the affected people. FMF is not evenly distributed, whether in countries where it is common, such as Turkey [31], Armenia [42], Lebanon [28,43], or Iran [44], or in those where it is classified as a rare disease [18]. In 2008, Papadopoulos et al. showed that the distribution pattern of *MEFV* mutations were not uniform and that their presence is more FMF-related in Eastern than in Western European countries [45]. In 2014, Ozen et al., using the Eurofever Registry of auto-inflammatory diseases [46], studied the influence exerted by migrations, the environment, and genetics on the clinical expression of this disease. They concluded that patients from countries located in Eastern Mediterranean countries who emigrate to Europe present with a milder disease than do those who remain in those countries. Based on this, they reflected on the effect of migration, lifestyle, and environment on the phenotypic expression of the disease. In addition, they described that the factors that significantly heightened the severity and risk of amyloidosis were: country of residence (living in an Eastern Mediterranean country); the presence of the M694V mutation (more common in patients from Eastern Mediterranean countries); and family history of FMF [46,47]. Although estimating the worldwide prevalence of *MEFV* mutations is difficult, a meta-analysis of the genetic population of FMF in 14 different affected Mediterranean populations described the mean overall carrier rate as 0.186 [45]. Different mutations, such as M694V, E148Q, K695R, and M680I, have been described in the Spanish population with FMF [16,19,22]. Hence, a greater number of admissions could be expected in the provinces where the M694V mutation was more prevalent. Apart from this, differences between hospitals in the registration and coding of discharges, as well as those between the different Spanish provinces in the budgeting, health expenditure on, and care of people with rare diseases, could also account for the geographical variability found. In order to avoid delays in the FMF diagnosis, there are some tools to guide physicians to the correct diagnosis, such as EULAR recommendations, the Eurofever/PRINTO classification criteria, and the Eurofever and Infevers websites [34,48,49].

When it comes to the limitations of this study, it should be noted that diagnostic complexity plus ICD coding changes together render the process of registration and unequivocal identification of this disease in health information systems difficult [50]. Even under the specific heading “Familial Mediterranean Fever” of ICD-9-CM code 277.31, three synonymous terms for FMF appear (benign paroxysmal peritonitis, familial paroxysmal polyserositis, and benign recurrent polyserositis) [51], along with a different, very low-prevalence entity of which case series’ have been described, i.e., hereditary amyloid nephropathy or hereditary renal amyloidosis [52,53]. In addition to the importance of making an appropriate differential diagnosis, a form of coding is needed that distinguishes between both entities, since they have different treatments and prognoses.

Heterogeneity in the processes of clinical diagnosis and registration of the disease could also affect the results reported, and, although the data provided by the MBDS are representative of the cases treated in Spain, it is possible that the number of FMF-related hospitalizations might nevertheless be underestimated. In this respect, FMF is a rare disease which would benefit from its possible inclusion in the Spanish Rare Disease Registry [24], something that would both contribute to its visibility and minimize the limitations brought by recent coding changes to its specific identification. 

## 5. Conclusions

In conclusion, this is the first study to explore the temporal and geographical variability of FMF in Spain. An increase in the hospitalizations of patients with FMF and interprovincial variability in the risk of such hospitalization was in evidence across the study period. While these findings enhance the visibility of this disease in the health system, facilitating health management and planning, to monitor it more accurately, data from a population-based registry would ideally be required.

The health system should implement the necessary training and tools for guiding the physicians to the correct diagnosis.

## Figures and Tables

**Table 1:** Characteristics of FMF-related hospitalizations: 2008–2015.

	N (%)			
	Men	Women	Overall	*p*-Values
TOTAL HOSPITAL DISCHARGES	502 (52.3)	458 (47.7)	960 (100)	
FMF DIAGNOSES				0.609
Main diagnosis	211 (51.3)	200 (48.7)	411 (42.8)	
Secondary diagnoses	291 (53)	258 (47)	549 (52.3)	
HOSPITAL DEPARTMENT				0.16
Internal Medicine	195 (38.8)	147 (32.1)	342 (35.6)	
Pediatrics	127 (25.3)	132 (28.8)	259 (26.9)	
Nephrology	27 (5.4)	33 (7.2)	60 (6.3)	
Digestive	27 (5.4)	25 (5.7)	52 (5.4)	
Surgery (general and digestive)	21 (4.2)	14 (3.1)	35 (3.6)	
Rheumatology	18 (3.6)	16 (3.5)	34 (3.5)	
Cardiology	13 (2.6)	5 (1.1)	18 (1.9)	
Emergencies	9 (1.8)	5 (1.1)	14 (1.5)	
Hematology	8 (1.6)	6 (1.3)	14 (1.5)	
Remaining specializations	57 (11.4)	75 (16.4)	132 (13.8)	
HOSPITAL DISCHARGE				0.178
Home	485 (96.6)	444 (97)	929 (96.8)	
Death	9 (1.8)	4 (0.9)	13 (1.4)	
Transfer to another hospital	6 (1.2)	6 (1.3)	12 (1.2)	
Voluntary registration	2 (0.4)	0 (0.0)	2 (0.2)	
Transfer to another social health center	0 (0.0)	2 (0.4)	2 (0.2)	
Other	0 (0.0)	2 (0.4)	2 (0.2)	
HOSPITAL READMISSIONS	98 (19.5)	84 (18.3)	182 (18.9)	0.641

**Table 2:** Hospitalizations of patients with FMF, 2008–2015: main diagnoses according to ICD-9-MC groups.

MAIN DIAGNOSES	N (%)		
	Men	Women	Overall
Endocrine, nutritional and metabolic diseases, and immunity disorders *	216 (43)	205 (44.8)	421 (43.9)
Diseases of the digestive system	52 (10.3)	33 (7.2)	85 (8.9)
Diseases of the respiratory system	43 (8.5)	33 (7.2)	76 (7.9)
Symptoms, signs, and ill-defined conditions	31 (6.2)	44 (9.6)	75 (7.8)
Diseases of the circulatory system	36 (7.2)	14 (3.1)	50 (5.2)
Diseases of the musculoskeletal system and connective tissue	24 (4.8)	23 (5)	47 (4.9)
Infectious and parasitic diseases	23 (4.6)	23 (5)	46 (4.8)
Diseases of the genitourinary system	20 (4)	23 (5)	43 (4.5)
Injury and poisoning	22 (4.4)	11 (2.4)	33 (3.4)
Complications of pregnancy, childbirth, and the puerperium	0 (0)	21 (4.6)	21 (2.2)
Neoplasms	11 (2.2)	8 (1.7)	19 (2)
Diseases of the nervous system and sense organs	6 (1.2)	8 (1.7)	14 (1.5)
Diseases of the blood and blood-forming organs	7 (1.4)	2 (0.5)	9 (0.9)
Diseases of the skin and subcutaneous tissue	3 (0.6)	6 (1.3)	9 (0.9)
Supplementary classification of factors that influence the state of health and contact with health services	3 (0.6)	3 (0.7)	6 (0.6)
Mental, behavioral, and neurodevelopmental disorders	4 (0.8)	0 (0)	4 (0.4)
Congenital anomalies	0 (0)	1 (0.2)	1 (0.1)
External causes of injury and supplemental classification	1 (0.2)	0 (0)	1 (0.1)
Certain conditions originating in the perinatal period	0 (0)	0 (0)	0 (0)
*p-value < 0.05*			

* FMF is included in this group.

**Table 3:** Standardized morbidity ratios (SMR) and 95% confidence intervals by province, both overall and by sex. Only those with statistically significant lower- or higher-than-expected SMRs after indirect standardization are shown.

PROVINCE	SMR 2008–2015 (95% CI)		
	Both Sexes	Men	Women
Very low risk (<0.50)			
Guipúzcoa		0 (0.00–0.49)	
Cordoba	0.12 (0.01–0.44)	0.24 (0.03–0.85)	0 (0.00–0.47)
Cáceres	0.12 (0.00–0.69)		0 (0.00–0.97)
Las Palmas	0.27 (0.10–0.59)	0.26 (0.05–0.76)	0.28 (0.06–0.81)
Vizcaya	0.31 (0.12–0.64)	0.42 (0.14–0.98)	0.19 (0.02–0.69)
Malaga	0.33 (0.17–0.59)	0.29 (0.09–0.67)	0.38 (0.14–0.82)
Cadiz	0.46 (0.24–0.80)		0.24 (0.05–0.7)
Low risk (<1.00)			
Granada	0.53 (0.25–0.97)		0.22 (0.03–0.79)
Zaragoza			0.11 (0.00–0.61)
La Coruña			0.29 (0.06–0.85)
Jaén			0.15 (0.00–0.86)
Madrid	0.69 (0.55–0.84)		0.60 (0.43–0.83)
Asturias		0.19 (0.02–0.68)	
Ciudad Real		0 (0.00–0.66)	
High risk (>1.00)			
Barcelona	1.37 (1.16–1.6)	1.42 (1.14–1.76)	1.30 (1.06–1.65)
Valencia	1.39 (1.08–1.74)	1.72 (1.26–2.28)	
Islas Baleares			1.97 (1.22–3.01)
Alicante	1.62 (1.24–2.07)		2.50 (1.82–3.35)
Murcia	1.72 (1.29–2.24)	1.91 (1.30–2.71)	
Huelva	1.94 (1.20–2.97)		2.52 (1.34–4.30)
Castellón	2.00 (1.28–2.97)	2.34 (1.31–3.87)	
Very high risk (>2.00)			
Huesca			2.97 (1.09–6.47)
Guipúzcoa			2.12 (1.16–3.56)
Valladolid	2.20 (1.39–3.29)		3.67 (2.18–5.80)
Segovia	3.15 (1.51–5.80)		3.41 (1.10–7.95)
Toledo	3.61 (2.69–4.73)	5.59 (4.05–7.53)	
La Rioja	4.06 (2.65–5.95)		8.35 (5.40–12.33)
95% CI: 95% confidence interval.			

## Data Availability

Not applicable.
